# Intracardiac vs. transesophageal echocardiography for guiding transcatheter closure of interatrial communications: a systematic review and meta-analysis

**DOI:** 10.3389/fcvm.2023.1082663

**Published:** 2023-05-05

**Authors:** Qingsu Lan, Fengchao Wu, Xudong Ye, Shaohua Wang, Jingquan Zhong

**Affiliations:** ^1^ National Key Laboratory for Innovation and Transformation of Luobing Theory; The Key Laboratory of Cardiovascular Remodeling and Function Research, Chinese Ministry of Education, Chinese National Health Commission and Chinese Academy of Medical Sciences; Department of Cardiology, Qilu Hospital of Shandong University, Jinan, China; ^2^ Cardiology Department, Shanxi Provincial People's Hospital, Xi'an, China; ^3^ The First Clinical Medical College, Lanzhou University, Lanzhou, China; ^4^ Clinical Medical College, Dali University, Dali, China; ^5^ Department of Cardiology, Qilu Hospital (Qingdao), Cheeloo College of Medicine, Shandong University, Qingdao, China

**Keywords:** atrial septal defect, patent foramen ovale, transesophageal echocardiography, intracardiac echocardiography, congenital, congenital heart diasease

## Abstract

**Background:**

Transcatheter closure of atrial septal defect (ASD) and patent foramen ovale (PFO) is an established practice, and it requires monitoring and guidance. Both transoesophageal echocardiography (TEE) and intracardiac echocardiography (ICE) can be used as guidance tools. However, the use of ICE and TEE in structural heart disease is controversial and the advantages and disadvantages of both for ASD and PFO closure need to be investigated. We did a systematic review and meta-analysis to compare the efficacy and safety of TEE and ICE for guiding transcatheter closure of ASD and PFO.

**Methods:**

A systematic search of Embase, PubMed, Cochrane library, Web of Science was conducted from inception to May 2022. The outcomes of this study included average time for both fluoroscopy and the procedure, complete closure, length of stay at hospital and adverse events. This study was performed using mean difference (MD), relative risk (RR) and 95% confidence interval (CI).

**Results:**

The meta-analysis was conducted with a total of 11 studies, involving 4,748 patients were included in meta-analysis, including 2,386 patients in the ICE group and 2,362 patients in the TEE group. The results of the meta-analysis showed that compared with TEE, ICE was shorter in time both fluoroscopy [MD: −3.72 (95%CI: −4.09 to −3.34) minutes, *P* < 0.00001] and the procedure [MD: −6.43 (95%CI: −7.65 to −5.21) minutes, *P* < 0.00001], shorter length of stay at hospital [MD = −0.95 (95% CI = −1.21 to −0.69) days, *P* < 0.00001], lower incidence of adverse events (RR = 0.72, 95%CI: 0.62 to 0.84, *P* < 0.0001), and the arrhythmia (RR = 0.50, 95% CI = 0.27 to 0.94, *P *= 0.03) and vascular complications (RR = 0.52, 95%CI = 0.29 to 0.92, *P *= 0.02) in ICE group were lower than those in TEE group. No significant difference in complete closure was found between ICE and TEE (RR = 1.00, 95% CI = 0.98 to 1.03, *P *= 0.74).

**Conclusion:**

Under the premise of ensuring successful rate of complete closure, ICE can shorten time between fluoroscopy and procedure and length of stay at hospital, and there was no increase in adverse events. However, more high-quality studies are needed to confirm the benefits of using ICE in ASD and PFO closure.

## 1. Introduction

Atrial septal defect (ASD), one of the most common congenital heart diseases (CHDs) in adulthood, accounts for 25%–30% of newly diagnosed CHDs, which can cause systemic circulation blood to flow into the pulmonary circulation (1). However, the majority of ASD patients remain asymptomatic through infancy and early childhood. Even those with a large left-to-right shunt might not go undiagnosed until the onset of significant symptoms in adulthood (2). Patients with ASD are at risk of developing a range of complications as time goes on, such as arrhythmias, right heart failure, paradoxical embolism, occasionally pulmonary hypertension and even higher mortality rate compared to normal population (3, 4). The first catheter closure of ASD was reported in 1976 (5). Transcatheter intervention is the common treatment option when technically feasible. Like ASD, patent foramen ovale (PFO) is common cardiovascular disease. Although the relationship between PFO and cryptogenic stroke remains controversial, some studies have confirmed the efficacy and safety of PFO closure (6–9).

Both ASD and PFO closure are increasingly common interventional procedures, which are well-established option for both ASD and PFO, while a certain risk of severe complications remains (10). The choice of echocardiographic monitoring is crucial to avoid several complications of transcatheter closure. During the procedure, interatrial communications could be assessed by transesophageal echocardiography (TEE) with defect size and position, proximity to surrounding structures, rim morphology, and final device positioning (11). However, some preparations during TEE examination, such as fasting and drinking forbidden, may cause discomfort to the patient, and potential risks should be considered, including esophageal injury, general anesthesia (GA) and tracheal intubation, which may lead to death when complications occur. In addition, the imaging of TEE is suboptimal for the lower part of the atrial septum (12, 13). With the continuous updating and rapid development of interventional equipment, intracardiac echocardiography (ICE) has been used as guidance during transcatheter closure. The clinical application of ICE dates back to the 1960s (14). Compared with TEE, ICE does not need GA and does not cause esophageal injury, which can avoid the occurrence of possible complications (15, 16). ICE has been used to guide numerous invasive cardiac procedures and interventions, especially in ASD and PFO (17–20). However, some studies concluded that ICE was used to patients with complex lesions and should not be used routinely (20–22). Therefore, we performed a meta-analysis to evaluate if the use of ICE is superior to TEE during ASD and PFO closure.

## 2. Materials and methods

### 2.1. Studies selection

A systematic search of Web of Science, Embase, PubMed and Cochrane library was conducted from inception to May 2022. Cohort studies on the comparison of ICE and TEE in transcatheter closure of ASD and PFO were collected. The search was conducted by combining subject words and free words, and adjusted according to the characteristics of each database. The articles' reference lists were retrieved to supplement the relevant information. No filters or language restrictions used. The search strategy included the main search terms “Heart Defects, Congenital”, “Atrial septal defect”, “Patent Foramen Ovale”, “intracardiac echocardiography”. Articles were subjected to review by two researchers indepen dently after the title and abstract screening. Disagreements were resolved through adjudication by a third researcher.

### 2.2. Inclusion and exclusion criteria

Studies which met the inclusion criteria were eligible for the meta-analysis: (1) Study type was cohort study; (2) Patients with a confirmed diagnosis of ASD or PFO were included; (3) Comparison of percutaneous transcatheter closure of ASD or PFO with different guidance tools; (4) One or more of the following outcome indicators were in the included article: average time for both fluoroscopy and the procedure, complete closure, length of stay at hospital and adverse events.

Exclusion criteria are as follows: (1) Duplicated articles, reviews, case reports, commentaries, editorials and conference abstract; (2) No data, incomplete or incorrect data; (3) Non- relevant outcomes.

### 2.3. Data collection and quality assessment

We extracted the following data: (1) The first author, year of publication, country, type of study, participation, sample size, gender, age, assistance by fluoroscopy, follow-up, outcome assessment; (2) Intervention measures: the guidance tools of different groups; (3) Outcomes: average time for both fluoroscopy and the procedure, complete closure, length of stay at hospital and adverse events. Two researchers accessed the included studies and the quality of the articles, and another researcher would resolve by discussion to reach consensus if there were disagreement. We used Newcastle-Ottawa Quality Assessment Scale (NOS) to evaluate the risk of deviation of the included studies. NOS is composed of three aspects (selection of participants, comparability of research groups and results) with a maximum score of 9 points. NOS score of six or higher was high-quality studies.

### 2.4. Data synthesis and analysis

Statistical analysis was performed using Review Manager software. Dichotomous data were present as relative risks (RR) with 95% confidence intervals (CI) and continuous data were expressed as mean difference (MD) with 95% CI. All effective outcomes were evaluated for heterogeneity by chi-square test and it was quantified by I-squares values. I-squared <50% indicates the absence of heterogeneity and a fixed-effects model was used. Otherwise, the random effect model was applied for meta-analysis. The results were analysed by funnel plot to assess publication bias. Sensitivity analyses were performed to find sources of heterogeneity by removing each study from include studies to access the robustness of the results. *P*-value < 0.05 was considered statistically significance.

## 3. Results

**Figure 1:**
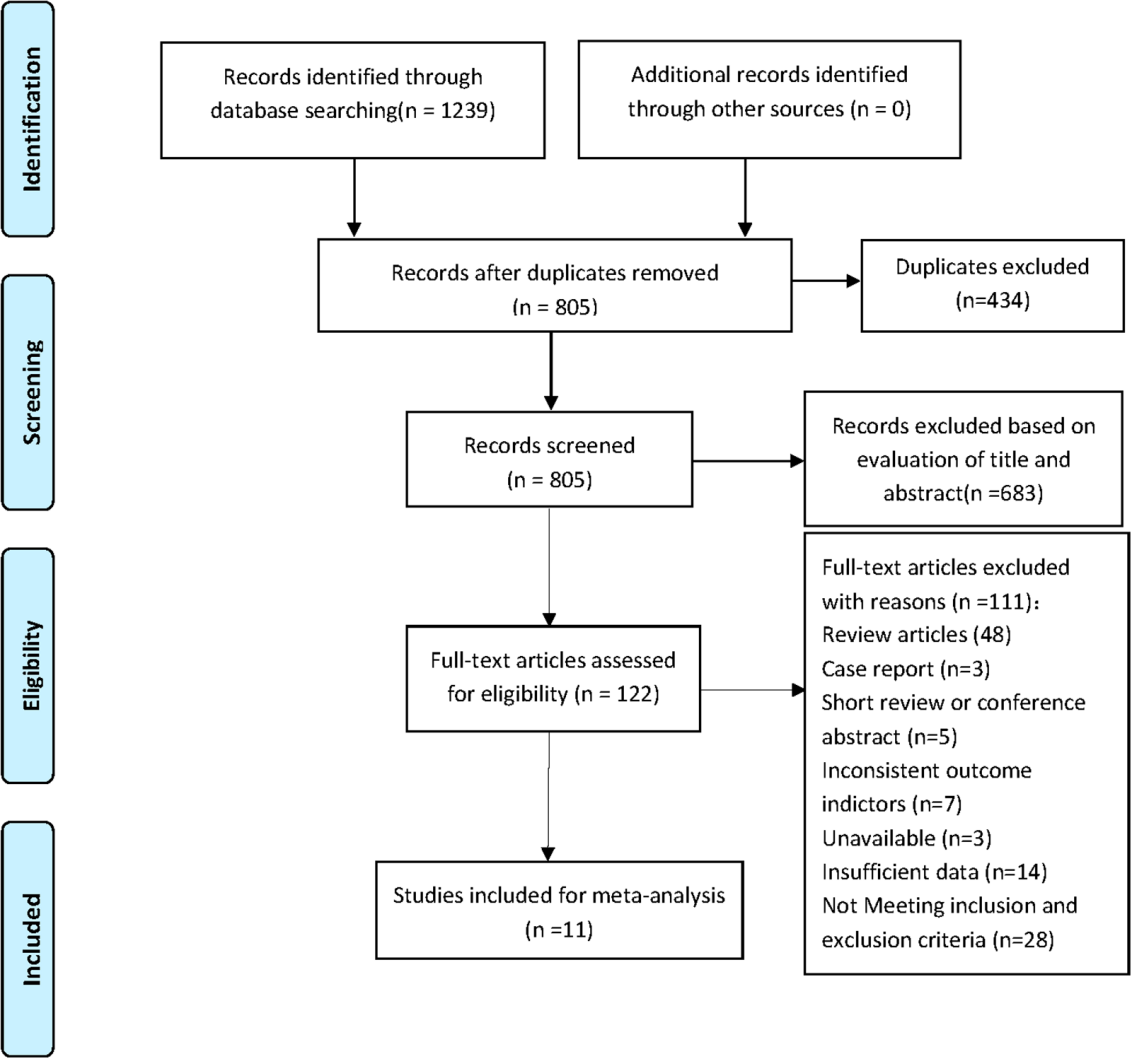
Flow diagram of the study search and selection process.

Two individual researchers retrieved for a total of 1,239 studies from Embase, Cochrane library, PubMed and Web of Science through the search strategy. There were 805 studies left after removing duplicate literature and 122 studies left after reading abstract and title. According to inclusion and exclusion criteria, 11 studies were finally included for meta-analysis. The NOS score of the included studies is 5–8, and there are 8 high-quality studies. Figure 1 shows the flow chart of screening.

A total of 11 studies (23–33) including 4,748 patients were included for this meta-analysis. There were 2,386 patients in the ICE group and 2,362 patients in the TEE group (Table 1). All included studies counted their sample size, age, female ratio, country and duration of follow-up. At the same time, assistance by fluoroscopy of the included studies was recorded in detail. All contents were summarized in Table 1.

**Table 1:** Main characteristics of the included studies.

First author	Year	Country	Type of study	Participation	Sample size (number) (ICE/TEE)	% Female (ICE/TEE)	Age (years) (ICE/TEE)	Assistance by fluoroscopy	Follow-up (months)	Outcome assessment	NOS Score
Zanchetta (23)	2004	Italy	Cohort study	ASD	31/124	73.4/77.4	42.5 ± 19.3 /40.5 ± 20.6	supplemented by fluoroscopy	12	complete closure, PT and FT	7
Bartel (21)	2003	Germany	Cohort study	ASD, PFO	22/22	NA	NA	ICE: supplemented by fluoroscopy as needed TEE: primarily guided by fluoroscopy	-	complications, PT and, FT	5
Boccalandro (22)	2004	American	Cohort study	ASD, PFO	21/21	57.1/5 2.3	50 ± 16/48 ± 16	ICE/TEE and quantitative fluoroscopy	-	complete closure, complications	6
Bartel (24)	2005	Germany	Cohort study	ASD, PFO	50/30	45^a^	46 ± 13^a^	ICE: supplemented by fluoroscopy, TEE: guided by fluoroscopy and additionally monitored by TEE	6	complications, PT and, FT	6
Chien (25)	2008	China	Cohort study	ASD	128/142	NA	20.9 ± 16.8/15.3 ± 14.9	supplemented by fluoroscopy	-	FT, PT, Defect size, complications	7
Kim (26)	2012	korea	Cohort study	ASD	323/237	0.34/0.73	22 ± 7.7/23.0 ± 4.0	supplemented by fluoroscopy	-	FT,PT, Success rate, complications, device size	7
Shimizu (27)	2013	Japan	Cohort study	ASD	51/41	56.9 / 63.4	48.4 ± 48.0/44.9 ± 59.9^b^	ICE or TEE combined with fluoroscopy before release	11.2	Success rate, complications, Catheterization laboratory time	8
Alqahtani (29)	2017	American	Cohort study	ASD, PFO	1,659/1,659	42.8/42.9	53 ± 16/53 ± 16	NA	-	MACCE,, complications, length of stay, cost	7
Yamano (30)	2020	Japan	Cohort study	ASD	53/14	NA	47 ± 20/26 ± 22	supplemented by fluoroscopy	40.0 ± 31.3^b^	PT, length of stay, cost, doses of sedatives, complications	7
Moon (31)	2020	Korea	Cohort study	PFO	25/49	28/33	57 ± 7/47 ± 10	supplemented by fluoroscopy	-	FT, PT, radiation dose, length of stay,complications	5
Zhao (28)	2015	China	Cohort study	ASD	23/23	48/48	46.9 ± 18.2/41.8 ± 18.7	supplemented by fluoroscopy	-	Success rate, complications, PT, FT	5

^a^ Variables for all included patients.

^b^ The original study only provided the Median and Inter-Quartile Range. After numerical conversion according to the method of Hozo etc (32), the Mean^ ± ^Standard Deviation was obtained.

ASD, atrial septal defect; PFO, patent foramen ovale; ICE, intracardiac echocardiography; TEE, transoesophageal echocardiography; PT, procedure time (minutes); FT, fluoroscopy time (minutes); MACCE, major adverse cardiac and cerebrovascular event; NA, not available;.

### 3.1. Average time for both fluoroscopy and the procedure

**Figure 2:**
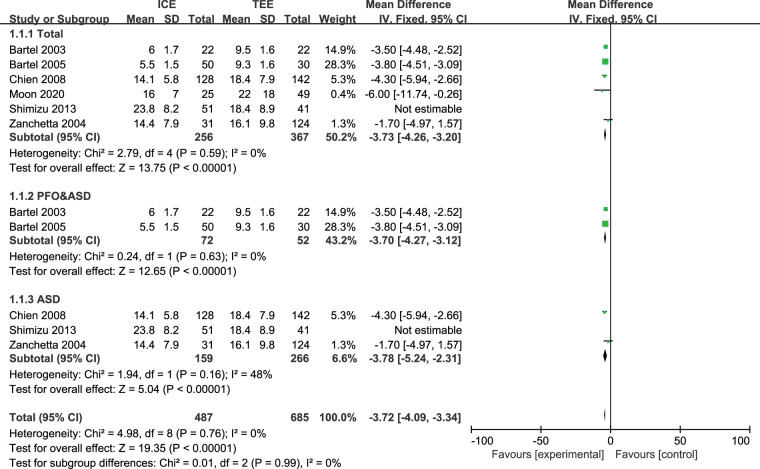
Forest plot for the meta-analysis of FT (mintues).

**Figure 3:**
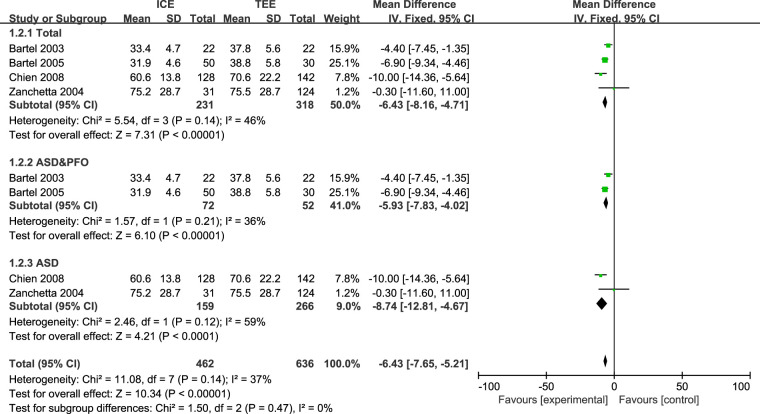
Forest plot for the meta-analysis of PT (mintues).

Six studies (23, 25–27, 29, 33) reported fluoroscopy time (FT) with a total of 623 patients and four studies (23, 25–27) reported procedure time (PT) with a total of 598 patients. Due to the high heterogeneity (FT: *I*^2 ^= 70%, *P* = 0.0004), we conducted sensitivity analysis in order to explore the sources of heterogeneity. After we removed the Schimizu 2013 (29) study, heterogeneity across the studies was relatively low (FT: *I*^2 ^= 0%, *P* = 0.76, PT: *I*^2 ^= 37%, *P *= 0.14). Results indicated that times for both fluoroscopy [MD: −3.72 (95%CI: −4.09 to −3.34) minutes, *P *< 0.00001, Figure 2] and the procedure [MD: −6.43 (95%CI: −7.65 to −5.21) minutes, *P* < 0.00001, Figure 3] were shorter in ICE group than in TEE group. The subgroup analysis results of ASD patients were consistent with the overall results (FT (total): MD: −3.73 [95%CI: −4.26 to −3.20] minutes, *P *< 0.00001, FT(PFO&ASD): MD: −3.70 [95%CI: −4.27 to −3.12] minutes, *P *< 0.00001, FT(ASD): MD: −3.78 [95%CI: −5.24 to −2.31] minutes, *P *< 0.00001, Figure 2; PT (total): MD: −6.43 [95%CI: −8.16 to −4.71] minutes, *P *< 0.00001, PT(PFO&ASD): MD: −5.93 [95%CI: −7.83 to −4.02] minutes, *P *< 0.00001, PT(ASD): MD: −8.74 [95%CI: −12.81 to −4.67] minutes, *P *< 0.00001, Figure 3). In addition, we would like to conduct further subgroup analysis on patients with PFO, but the data were limited for subgroup analysis.

### 3.2. Complete closure

**Figure 4:**
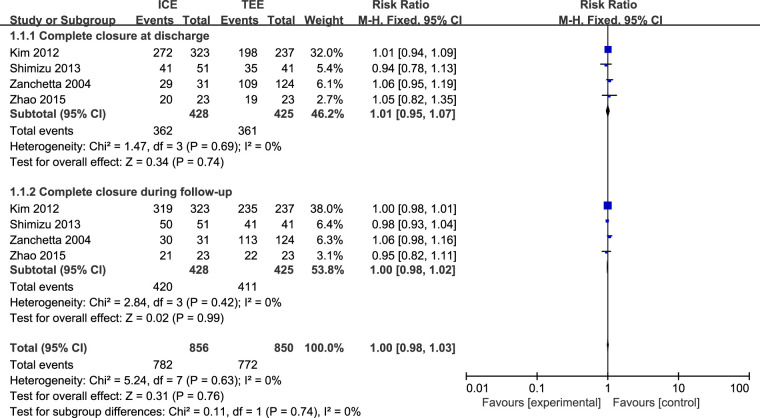
Forest plot for the meta-analysis of complete closure.

Four studies (25, 28–30) including 825 patients evaluated complete closure. No statistical heterogeneity was found among the studies (*I*^2 ^= 0%, *P *= 0.63). There was no difference in complete closure between ICE group and TEE group in the pooled results (RR = 1.00, 95% CI = 0.98 to 1.03, *P *= 0.74). A subgroup analysis was performed and no significant difference was found between ICE group and TEE group in complete closure at discharge (RR = 1.01, 95% CI = 0.95 to 1.07; *P *= 0.74) and follow-up (RR = 1.00, 95% CI = 0.98 to 1.02; *P *= 0.99, Figure 4). All included patients were all ASD patients, and subgroup analysis by disease type was not available.

### 3.3. Length of stay at hospital

**Figure 5:**
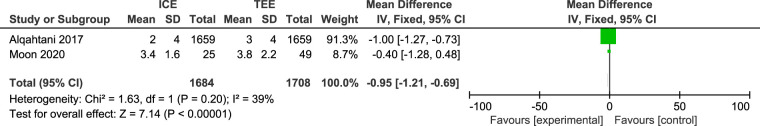
Forest plot for the meta-analysis of the length of hospitalization (days).

Two studies (31, 33) including 3,392 patients reported the length of stay at hospital, heterogeneity among the studies was relatively low (*I*^2 ^= 39%, *P *= 0.20). Compared to TEE for ASD and PFO, ICE can reduce the length of stay at hospital [MD = −0.95 (95% CI = −1.21 to −0.69) days; *P *< 0.00001, Figure 5].

### 3.4. Adverse events

**Figure 6:**
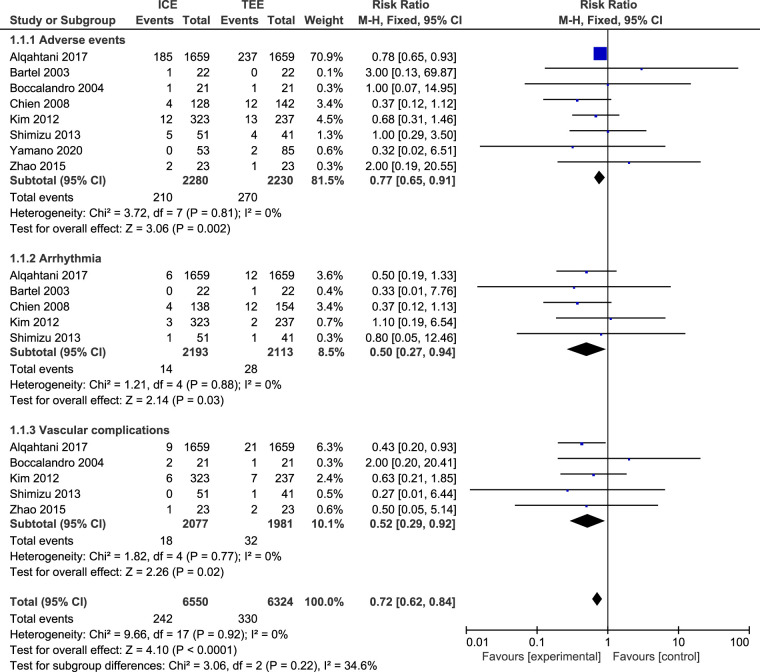
Forest plot for the meta-analysis of adverse events.

Eight studies (23, 24, 27–32) including 4,510 patients reported adverse events, we selected a fixed-effect model for meta-analysis because I-squared was less than 50% (*I*^2 ^= 0%, *P *= 0.92). The adverse events of TEE were significantly higher than that in the ICE (RR = 0.72, 95%CI:0.62 to 0.84, *P *= 0.002, Figure 6). We further conducted subgroup analysis on adverse events and we found that the arrhythmia (RR = 0.50, 95%CI = 0.27 to 0.94, *P *= 0.03, Figure 6) and vascular complications (RR = 0.52, 95%CI = 0.29 to 0.92, *P *= 0.02, Figure 6) in ICE group were lower than those in TEE group.

### 3.5. Publication bias analysis

**Figure 7:**
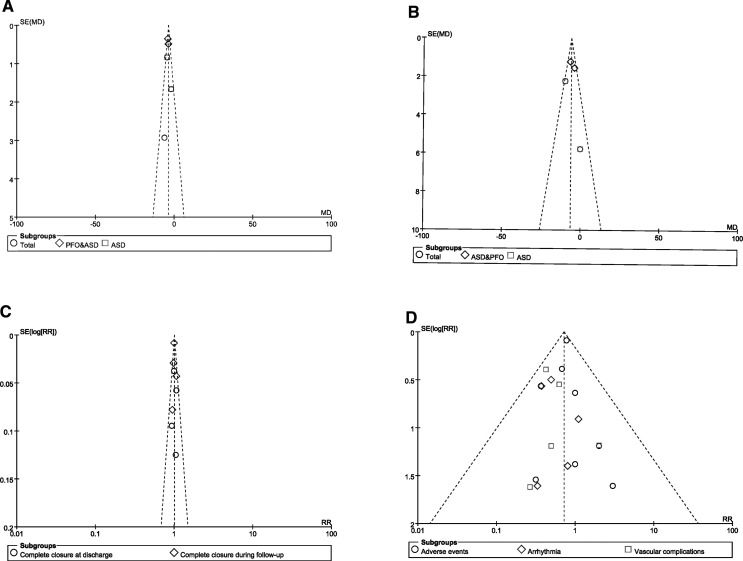
Funnel plot of publication bias. (**A**) Funnel plots for the meta-analysis of the FT; (**B**) Funnel plots for the meta-analysis of the PT; (**C**) Funnel plots for the meta-analysis of complete closure; (**D**) Funnel plots for the meta-analysis of adverse events.

We drew the funnel plots to detect the potential risk of publication bias, and the funnel plots showed that the scattered points distribution were symmetrical from the point of view of the geometry, indicating that the risk of bias was low for the studies included in our meta-analysis (Figure 7).

## 4. Discussion

We evaluated the efficacy and safety of ICE as a guidance tool for ASD and PFO closure. On this basis, we further clarified the impact of ICE guidance on FT, PT and the length of hospitalization. The findings of this article are as follows: ① Compared with TEE, the success rate of complete occlusion using ICE as guidance tool is similar to that of TEE. Further subgroup analysis showed that there was no difference between the two groups at discharge and follow-up; ② Transcatheter closure of ASD and PFO under ICE guidance can shorten FT, PT and the length of stay at hospital; ③ The use of ICE may decrease the incidence of adverse reactions compared with TEE.Although our subgroup analysis did not significantly increase the incidence of arrhythmia and vascular related adverse events, the use of a second catheter may lead to the increase of catheter-related complications.

With the use of safe and effective sealing devices and the advances in interventional tools, the non-surgical treatment of ASD and PFO has become possible. Percutaneous closure of interatrial communications has become the preferred treatment for most patients. Compared with surgery, interventional treatment has faster recovery and less trauma, but it is under the guidance of fluoroscopy during procedure. Radiation can cause plenty of inevitable side effects to physiological functions of patients and physicians, which results in damage to the hematopoietic function of bone marrow, interference with normal function of thyroid and potential risks of developing various cancers (12). Therefore, FT and PT should be as short as possible. It is presented in this study that FT and PT can be shortened under ICE guidance during transcatheter closure of ASD and PFO. The main reasons for the time reduction are as follows: GA not being used can significantly reduce PT. As for the FT, some studies point to higher image quality with ICE than TEE, fluoroscopy and transthoracic echocardiography for accurate evaluation of intracardiac structures, which might save time (27, 34).

Classically, TEE has been used in ASD and PFO closure with fluoroscopy for procedural guidance. However, TEE guidance requires the patient to be under GA with or without endotracheal intubation (12). Complications caused by GA are less, but it is more serious when it occurs. While ICE not only eliminates the need for GA, but also provides clearer and more accurate in assessing defects and the observation of adjacent structures (35). However, we did not find the advantage of using ICE guidance in the success rate of closure. Compared with TEE, some scholars are worried that the use of ICE will increase the hospitalization cost, but ICE guidance to ASD and PFO closure did not increase the global cost (29). We consider the following reasons: first, during GA, anesthesiologists are required to monitor the fluctuation of heart rate, blood pressure, oxygen saturation continuously, however, if ICE is used as guidance tool during operation, GA is not required and relevant physician, drugs and device support for GA is no longer needed; Second, ICE guidance does not require an additional echocardiographer and only cardiologist can perform the procedure (12, 36, 37); Finally, it is found in this study that the length of hospitalization in the ICE group is shortened. The cost of TEE can offset the additional cost of the ICE catheter. Previous studies showed that similar costs for hospital and physician charges using ICE or TEE (US dollars 34,861 ± 43,759 vs. US dollars 32,812 ± 2,656, *P* = 0.107) (38). Taking into account labour, additional individual requirements and anaesthetic costs, the total costs of ICE and TEE might be comparable (39). Although the relevant studies in Europe and the United States mentioned above have not found the cost increase caused by the use of ICE, reusability of the catheter is limited and health insurance agencies in many countries do not cover the costs of ICE catheters, which is a heavy burden for patients. In addition, ICE as a monitoring tool will increase the need for an additional venous sheath,and it can cause the danger of potentially provoking transient atrial arrhythmias (40, 41).This is not consistent with the results of this article.

The limitations of this study include: ① The relatively small sample size of included studies may affect the accuracy of the results; ② Different studies include different patients among the included articles. For example, some studies only include ASD patients, some studies only include PFO patients, while some studies include both ASD and PFO patients; ③ Different research institutes use different equipment. For example, some research centers, like Zhao 2015 (30), used Philips echocardiography equipment, while others use vivid I (32). The difference may lead to the possibility of bias.

## 5. Conclusion

In conclusion, our study suggests that ICE can be used safely and effectively in transcatheter closure of ASD and PFO, which provides shorter FT and PF, shorter hospital stay, and decrease in adverse events and no significant difference in complete closure. In the future, we still require more large-scale high-quality studies in order to further confirm the safety and efficacy of ICE for image guidance in ASD and PFO.

## Data Availability

The original contributions presented in the study are included in the article/**Supplementary Material**, further inquiries can be directed to the corresponding author.
