# Supplementary material for: Intracardiac vs. transesophageal echocardiography for guiding transcatheter closure of interatrial communications: a systematic review and meta-analysis
Source: Front Cardiovasc Med. 2023 May 5;10:1082663. doi: 10.3389/fcvm.2023.1082663 (PMC10198467; doi:10.3389/fcvm.2023.1082663)
Supplement: Supplementary file 1 [file Table1.docx]

Intracardiac Echocardiography versus Transesophageal Echocardiography for Guiding Transcatheter Closure of Atrial Septal Defect and Patent Foramen Ovale: a Systematic Review and Meta-analysis

Supplementary Table 1. The search strategies for all databases.

| Pubmed | Search terms |
| --- | --- |
| #1 | (atrial septal defect[Title/Abstract]) OR (ASD[Title/Abstract) OR (Patent foramen ovale[Title/Abstract]) OR (PFO[Title/Abstract]) OR (ventricular septal defect[Title/Abstract]) OR (VSD[Title/Abstract]) OR (pulmonary stenosis[Title/Abstract]) OR (patent ductus arteriosus[Title/Abstract]) OR (tetralogy of Fallot[Title/Abstract]) OR (TOF[Title/Abstract]) OR (coarctation[Title/Abstract]) OR (transposition of the great arteries[Title/Abstract]) OR (aortic stenosis[Title/Abstract]) OR (congenital heart disease[Title/Abstract]) OR (congenital heart defect[Title/Abstract]) OR (congenital heart malformation[Title/Abstract]) OR (congenital heart anomalies[Title/Abstract]) OR (congenital cardiac disease[Title/Abstract]) OR (congenital cardiac defect[Title/Abstract]) OR (congenital cardiac malformation[Title/Abstract]) OR (congenital cardiac anomalies[Title/Abstract]) OR (congenital cardiovascular disease[Title/Abstract]) OR (cardiovascular malformation[Title/Abstract]) OR (cardiovascular defect[Title/Abstract]) OR (cardiovascular anomalies[Title/Abstract]) |
| #2 | (intracardiac echocardiography[Title/Abstract]) OR (ICE[Title/Abstract]) OR (ICE-guided[Title/Abstract]) OR (intracardiac ultrasound[Title/Abstract]) OR (Ultra ICE[Title/Abstract]) OR (UltraICE[Title/Abstract]) OR (Soundstar[Title/Abstract]) OR (CartoSound[Title/Abstract]) OR (AcuNav[Title/Abstract]) |
| Combined set | #1 AND #2 |

| Cochrane | Search terms |
| --- | --- |
| #1 | (atrial septal defect):ab,ti,kw or (ASD):ab,ti,kw or (Patent foramen ovale):ab,ti,kw or (PFO):ab,ti,kw or (ventricular septal defect):ab,ti,kw or (VSD):ab,ti,kw or (pulmonary stenosis):ab,ti,kw or (patent ductus arteriosus):ab,ti,kw or (tetralogy of Fallot):ab,ti,kw or (TOF):ab,ti,kw or (coarctation):ab,ti,kw or (transposition of the great arteries):ab,ti,kw or (aortic stenosis):ab,ti,kw or (congenital heart disease):ab,ti,kw or (congenital heart defect):ab,ti,kw or (congenital heart malformation):ab,ti,kw or (congenital heart anomalies):ab,ti,kw or (congenital cardiac disease):ab,ti,kw or (congenital cardiac defect):ab,ti,kw or (congenital cardiac malformation):ab,ti,kw or (congenital cardiac anomalies):ab,ti,kw or (congenital cardiovascular disease):ab,ti,kw or (cardiovascular malformation):ab,ti,kw or (cardiovascular defect):ab,ti,kw or (cardiovascular anomalies):ab,ti,kw |
| #2 | (intracardiac echocardiography):ab,ti,kw or (ICE):ab,ti,kw or (ICE-guided):ab,ti,kw or (intracardiac ultrasound):ab,ti,kw or (Ultra ICE):ab,ti,kw or (UltraICE):ab,ti,kw or (Soundstar):ab,ti,kw or (CartoSound):ab,ti,kw or (AcuNav):ab,ti,kw |
| Combined set | #1 And #2 |

| Embase | Search terms |
| --- | --- |
| #1 | 'atrial septal defect':ab,ti or 'ASD':ab,ti or 'Patent foramen ovale':ab,ti or 'PFO':ab,ti or 'ventricular septal defect':ab,ti or 'VSD':ab,ti or 'pulmonary stenosis':ab,ti or 'patent ductus arteriosus':ab,ti or 'tetralogy of Fallot':ab,ti or 'TOF':ab,ti or 'coarctation':ab,ti or 'transposition of the great arteries':ab,ti or 'aortic stenosis':ab,ti or 'congenital heart disease':ab,ti or 'congenital heart defect':ab,ti or 'congenital heart malformation':ab,ti or 'congenital heart anomalies':ab,ti or 'congenital cardiac disease':ab,ti or 'congenital cardiac defect':ab,ti or 'congenital cardiac malformation':ab,ti or 'congenital cardiac anomalies':ab,ti or 'congenital cardiovascular disease':ab,ti or 'cardiovascular malformation':ab,ti or 'cardiovascular defect':ab,ti or 'cardiovascular anomalies':ab,ti |
| #2 | 'intracardiac echocardiography':ab,ti or 'ICE':ab,ti or 'ICE-guided':ab,ti or 'intracardiac ultrasound':ab,ti or 'Ultra ICE':ab,ti or 'UltraICE':ab,ti or 'Soundstar':ab,ti or 'CartoSound':ab,ti or 'AcuNav':ab,ti |
| Combined set | #1 And #2 |

| Web of science | Search terms |
| --- | --- |
| #1 | TI=(atrial septal defect or ASD or Patent foramen ovale or PFO or ventricular septal defect or VSD or pulmonary stenosis or patent ductus arteriosus or tetralogy of Fallot or TOF or coarctation or transposition of the great arteries or aortic stenosis or congenital heart disease or congenital heart defect or congenital heart malformation or congenital heart anomalies or congenital cardiac disease or congenital cardiac defect or congenital cardiac malformation or congenital cardiac anomalies or congenital cardiovascular disease or cardiovascular malformation or cardiovascular defect or cardiovascular anomalies) |
| #2 | TI=(intracardiac echocardiography or ICE or ICE-guided or intracardiac ultrasound or Ultra ICE or UltraICE or Soundstar or CartoSound or AcuNav) |
| Combined set | #1AND #2 |
